# DBS of the ANT for refractory epilepsy: A single center experience of seizure reduction, side effects and neuropsychological outcomes

**DOI:** 10.3389/fneur.2023.1106511

**Published:** 2023-03-09

**Authors:** Karmele Olaciregui Dague, Juri-Alexander Witt, Randi von Wrede, Christoph Helmstaedter, Rainer Surges

**Affiliations:** Department of Epileptology, University Hospital Bonn, Bonn, Germany

**Keywords:** deep brain stimulation, refractory epilepsy, cognition, side effects, neuropsychological assessment

## Abstract

**Objective:**

Evaluation of the antiseizure efficacy, side effects and neuropsychological effects of Deep brain stimulation (DBS) of the anterior nucleus of the thalamus (ANT). ANT-DBS is a treatment option for patients with difficult-to-treat epilepsy. Though several works outline the cognitive and/or mood effects of ANT-DBS for the treatment of epilepsy, data on the intersection between antiseizure efficacy, cognitive and undesired effects are scarce.

**Methods:**

We retrospectively analyzed the data of our cohort of 13 patients. Post-implantation seizure frequencies were measured at 6 months, 12 months and last follow-up, as well as averaged throughout follow-up. These values were then compared with mean seizure frequencies in the 6 months before implantation. To address acute cognitive effects of DBS a baseline assessment was performed after implantation and before stimulation, and a follow-up assessment was conducted under DBS. The long-term effects of DBS on cognition were assessed by comparing the preoperative neuropsychological profile with a long-term follow-up under DBS.

**Results:**

In the entire cohort, 54.5% of patients were responders, with an average seizure reduction of 73.6%. One of these patients achieved temporary seizure freedom and near-total seizure reduction during the entire follow-up. Seizure reduction of <50% was achieved in 3 patients. Non-responders suffered an average seizure increase of 27.3%. Eight of twenty-two active electrodes (36,4%) were off-target. Two of our patients had both electrodes implanted off-target. When removing these two patients from the analysis and averaging seizure frequency during the entire follow-up period, four patients (44.4%) were responders and three experienced a seizure reduction of <50%. Intolerable side effects arose in 5 patients, mostly psychiatric. Regarding acute cognitive effects of DBS, only one patient showed a significant decline in executive functions. Long-term neuropsychological effects included significant intraindividual changes in verbal learning and memory. Figural memory, attention and executive functions, confrontative naming and mental rotation were mostly unchanged, and improved in few cases.

**Significance:**

In our cohort, more than half of patients were responders. Psychiatric side effects seem to have been more prevalent compared to other published cohorts. This may be partially explained by a relatively high occurrence of off-target electrodes.

## Highlights

- Some multifocal and genetic epilepsies may respond well to ANT-DBS.
- Long-term neuropsychological outcomes are mixed.
- The most common side effects in our cohort were psychiatric.

## Introduction

Deep brain stimulation (DBS) of the anterior nucleus of the thalamus (ANT) is a treatment option for patients with difficult-to-treat epilepsy. ANT-DBS became an established therapy after the first (and only) prospective randomized controlled trial, the Stimulation of the Anterior Nucleus of the Thalamus for Epilepsy (SANTE) trial (1), showed promising results in its 3, 5 and 10-year follow-up studies (2, 3), with a 43% responder rate (≥50% reduction in seizure frequency) at 1 year (*n* = 99) and 74% at seven years (*n* = 50).

The antiseizure effects of ANT-DBS are thought to be based on the inhibition of seizure propagation through the thalamus (4) and modulation of the Circuit of Papez (5). Furthermore, increasing responder rates over the years have been attributed to long-term neuromodulation effects in neural networks.

Though several works (6, 7) outline the cognitive and/or mood effects of ANT-DBS for the treatment of epilepsy, data on the intersection between antiseizure efficacy, cognitive and undesired effects are scarce. We aimed to systematically evaluate the antiseizure efficacy, side effects and neuropsychological effects of ANT-DBS in epilepsy patients treated at our center.

## Methods

We retrospectively analyzed the data of our cohort of 13 patients, stereotactically and transventricularly implanted between 2012 and 2014, who underwent DBS for refractory epilepsy (Medtronic Activa PC Models 37601, 3787). Data on seizure reduction and side effects were complete in 11 of 13 patients. We defined refractory epilepsy according to the 2017 ILAE guidelines as non-responding to ≥2 anti-seizure medications (ASMs). Stimulation was usually initiated 3–5 weeks after implantation. We initially used the parameters described in the aforementioned landmark SANTE (1) study (Impulse width 90 μsec, Frequency 145 Hz, stimulation voltage 5.0 V, cycle: stimulation for 60 s every 5 min). Monopolar stimulation was used in all patients except the one patient with active VNS. When seizure frequency increased or did not decrease, changes in stimulation parameters were preferred to changes in ASM in order to better isolate the therapeutic effects of DBS. The preferred order of these changes was firstly changes in voltage (increase by 0.5–1 V), secondly changes in cycle speed (e.g. stimulation for 60 s every 3 min), and thirdly change into bipolar stimulation. These changes were carried out similarly in case of side effects, beginning with voltage decrease in 0.5 V steps. Nevertheless, ASM changes happened when deemed clinically necessary.

Post-implantation seizure reductions were expressed as percentages and interquartile ranges (IQR) and measured at 6 months, 12 months and last follow-up, as well as averaged throughout follow-up. These values were then compared with mean seizure frequencies in the 6 months before implantation. Seizure frequencies were assessed using seizure diaries. Seizure semiology was classified according to 2017 ILAE guidelines, based on our video-EEG (vEEG) recordings and descriptions by patients or witnesses. We analyzed the cohort of 11 patients in its entirety, and calculated the average seizure reduction during follow-up including only the patients who had at least one electrode on-target (*n* = 9).

In the current study we analyzed acute as well as long-term effects of DBS on cognition. To address acute cognitive effects a baseline assessment was performed after implantation and before initiating stimulation, and a follow-up assessment was conducted under DBS. The cognitive screening focused on attention and executive functions [EpiTrack^®^ (8)] and on verbal learning and episodic memory [short version of the Verbaler Lern- und Merkfähigkeitstest, VLMT (9)]. To analyze the long-term effects of DBS on cognition we compared the preoperative neuropsychological profile with a long-term follow-up under DBS. The neuropsychological assessment included tests on attention and executive functions [EpiTrack^®^ (8)], episodic long-term memory, i.e. verbal and figural learning and memory [VLMT (10) and a revised version of the Diagnosticum für Cerebralschädigung, DCS-R (11)], confrontative naming [Boston Naming Test, BNT (12)], and mental rotation [Leistungsprüfsystem, LPS subtest 7 (13)]. A mild impairment was defined as a performance lower than 1 standard deviation below the mean of the normative sample, a severe impairment as a performance lower than 2 standard deviations below the mean of the normative sample. Given the small sample size, we analyzed the frequencies of statistically significant intraindividual changes under DBS, employing reliable change indices (RCIs).

Follow-up duration during stimulation ranged from 9 to 111 months (average 51.5 months), and was ongoing until deactivation in all cases where deactivation occurred.

## Results

### Patient characteristics

#### Demographics

Age at implantation ranged from 22 to 50 (mean 35.5) years. Age at epilepsy onset was mostly in childhood and ranged from 4 to 24 (mean 14.5) years. Our cohort was 63.6% assigned female at birth (Table 1).

**Table 1:** Patient characteristics.

**Age at implantation**	**Sex assigned at birth**	**Etiology of epilepsy**	**Age at onset of epilepsy**	**ASM at implantation**	**VNS**	**Other epilepsy surgery**	**Side effects of DBS**	**Seizure reduction > 50% (avg during follow-up)**
45	f	GGE	12	5	n	n	Anxiety, right temporal headache after cycle increase	>50%
50	m	Multifocal encephalopathic epilepsy, etiology unknown	21	4	y (inactive)	n	None	Seizure increase
30	f	Dynamin-1 Mutation (etiology unkown at implantation)	8	3	y (inactive)	n	Polydipsia, functional movement disorder, FNES	< 50%
39	f	Unclear	12	4	n	y	None, wound problems	>50%
40	f	Perinatal left hemisphere substance defects, unclear origin	24	5	y (inactive)	y	Functional dysarthria and dysphagia	50%
29	f	Multifocal epilepsy, unclear origin	23	4	n	n	Initial concentration difficulties, immediate reversibility through voltage reduction. Paresthesias along cable	>50%
29	m	Post traumatic defect both superior frontal gyri	9	2	n	n	Delusional disorder	Seizure increase
22	f	Multifocal epilepsy, unclear origin	7	2	n	n	None	>50%
33	m	Suspected FCD left superior temporal gyrus	15	4	y (active during DBS)	y	None	< 50%
44	m	Post herpes encephalitis	25	4	y (inactive )	y	None	< 50%
30	f	Multifocal epilepsy, unclear origin	4	3	n	n	Burning dysesthesia around cable trajectory	>50%

#### Etiology

Among the 9 patients who experienced seizure reduction in the overall follow-up period, the etiology was most commonly unclear (5 patients, 55.5%), followed by structural origin. Five (55.5%) had multifocal epilepsy. Of the 2 patients with seizure increase, one had epilepsy of unknown etiology, the other structural epilepsy due to posttraumatic lesions. The patient who achieved seizure freedom had genetic generalized epilepsy. One of the responders with epilepsy of unknown origin, who underwent explantation due to polydipsia and the emergence of functional non-epileptic seizures, later underwent genetic testing that revealed a Dyamin-1 mutation suggesting a generalized encephalopathic epilepsy. Follow-up duration during stimulation ranged from 9 to 111 months (average 51.5 months), and is either ongoing, or continued until deactivation/explantation in all cases. Mean duration of stimulation (excluding the 3 patients with ongoing stimulation) was 52.6 months (range 10–97 months). When excluding the patient who underwent explantation before the 12 month follow-up mark due to intolerable side effects, all 7 patients underwent stimulation for at least 2 years and up to 6 years.

#### Anti-seizure medications

Patients were taking an average of 3.6 ASM at the time of implantation (range 2–5). Two patients underwent no changes in ASM during stimulation. Five patients, all of them DBS responders before the ASM change, underwent a substitution of one ASM due to side effects (exchange of one ASM for another), three patients were subject to more than one change in ASM (exchange, increase and/or reduction): two of these patients experienced a seizure increase during stimulation and underwent substitution and increase of one ASM, and one was a DBS responder (71% seizure decrease on average during entire follow-up) and underwent an exchange of one ASM due to side effects and a reduction of one ASM. One patient, who achieved temporary seizure freedom, was able to decrease the number of ASM. In two patients Perampanel was added, and in one Valproat was added, which may have influenced their psychiatric side effects, whether positively in the case of Valproate, or negatively in the case of Perampanel.

#### Concomitant VNS or prior surgeries

Presurgical evaluation including vEEG and/or stereo-EEG and 1.5 (due to VNS) or 3T MRI had taken place in all patients. Five of the patients in our cohort had undergone vagal nerve stimulation (VNS). Three of these were explanted at the time of initiating DBS, 1 remained implanted with an inactive VNS system, and 1 patient underwent simultaneous VNS (with constant stimulation parameters) and DBS stimulation. This patient suffered no side effects, and experienced a seizure reduction of 48.2% (average entire follow-up). One patient had undergone resective epilepsy surgery (lesionectomy of a left temporal cortical cavernoma) 12 years prior to implantation in another hospital. One patient had previously had a callosotomy 8 years prior.

#### Explantation and deactivation of DBS

**Figure 1:**
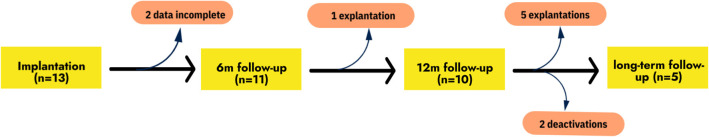
Study flowchart. Note: NPT is not detailed here.

Five patients remained implanted at the end of follow-up, and stimulation was ongoing in 3 patients (Figure 1). Causes for explantation or deactivation were: increased seizure frequency in 2 cases, side effects in 5 cases, and subjective insufficient seizure reduction in the remaining case.

### Seizure reduction and side effects

Data on seizure control were complete in all 11 patients. When averaging seizure frequency during the entire follow-up period, six patients (54.5%) were responders (achieved seizure reduction of ≥50%) (average 73.6% reduction, range 50–94.9%, interquartile range (IQR) 49.75). One of these patients achieved temporary seizure freedom and near-total seizure reduction during the entire follow-up. Seizure reduction of <50% was achieved in 3 patients (average seizure reduction of 42.7%). Among these nine patients with seizure reduction, the average seizure decrease during entire follow-up was 58.7% (range 36.5–100%, IQR 44.85). The 2 remaining patients had a seizure frequency increase ranging from 21.3 to 33.3% (average 27.3%). During follow-up, all patients underwent neuropsychological testing and were explicitly asked about side effects, including mood disorders.

At 6 months, 10 of the 11 patients (90.9%) reported seizure frequency reduction (7%-99% reduction in seizure frequency, average 53.8%, IQR 60.8). Of these, 4 patients (36.4%) reported a seizure frequency reduction of <50% (7%-43%, IQR 21.95). One patient had a 33.3% seizure increase. The remaining 6 patients (54.5%) were responders. At this point in time, 2 patients presented with side effects, both of psychiatric nature (one patient presented with new-onset daily functional non-epileptic seizures, one patient showed an exacerbation of previously existing depression).

At 12 months, the device had been deactivated in 1 patient, who previously had experienced a seizure reduction of >80%, due to side effects (intolerable paresthesias along subcutaneous cable trajectory, exacerbation of pre-existing depression). Eight of the 10 patients (80%) reported seizure frequency reduction (range 28.6–100%, average 78.8%, IQR 12.9), seven of them of >50%, with one patient reporting seizure freedom (61.9–100%, average 76.9%, IQR 11.8). The two remaining patients, one of whom had previously reported an increase in seizure frequency, had dramatic seizure increases of more than double the preoperative seizure frequency (145 and 233% increase), leading to changes in anti-seizure medication. At this point in follow-up, 5 patients reported side effects (emergence of delusions and episodic agitation, functional non-epileptic seizures, burning dysesthesia along the cable trajectory, functional dysarthria and dysphagia, functional polydipsia and emergence of functional non-epileptic seizures). All side effects were reported by patients with seizure reduction during stimulation, except in the case of emergence of delusions and episodic agitation in a patient with seizure increase.

At last point of follow-up (beyond 12 months) of the ten patients who remained implanted and undergoing stimulation, seven patients (70%) reported a decrease in seizure frequency (43.3–100%, IQR 35.3), six of them of >50% (58.3–99.8%, IQR 33.1). The three remaining patients, including the 2 patients who had suffered a significant seizure increase at 12 months, had returned to their preoperative seizure frequency. All of the previously reported side effects persisted, and ultimately led to explantation or deactivation. No patients reported suicidal ideation at any time point.

Two of our patients had both electrodes implanted off-target. They had a seizure reduction of 71% and 50% during the entire follow-up. Both showed side effects (functional non-epileptic seizures and functional dysarthria and dysphagia) that ultimately led to deactivation and/or explantation. When removing these two patients from the analysis and averaging seizure frequency during the entire follow-up period, four patients (44.4%) were responders (average reduction 86.7%, range 65.6–94.9, IQR 18.97) and three experienced a seizure reduction of < 50% (average reduction 42.6%, range 36.5–48.15, IQR 11.65). This cohort included the two patients with seizure increase as well as the patient that achieved temporary seizure freedom and near-total seizure reduction during the entire follow-up.

At their simplest, the outcomes in terms of side effects and seizure frequency in our cohort can be summarized as follows:

- one patient suffered a seizure increase and no side effects (etiology unknown)
- one patient suffered a seizure increase coupled with intolerable psychiatric side effects (structural posttraumatic etiology)
- four patients experienced a reduction in seizure frequency and no side effects (2 structural etiology, 1 genetic generalized epilepsy)
- one reported seizure reduction and tolerable side effects (etiology unknown)
- and the remaining four patients experienced seizure reduction coupled with intolerable side effects (etiologies unknown in 3 patients, structural 1, Dynamin-1 mutation 1).

#### Electrode placement

**Figure 2:**
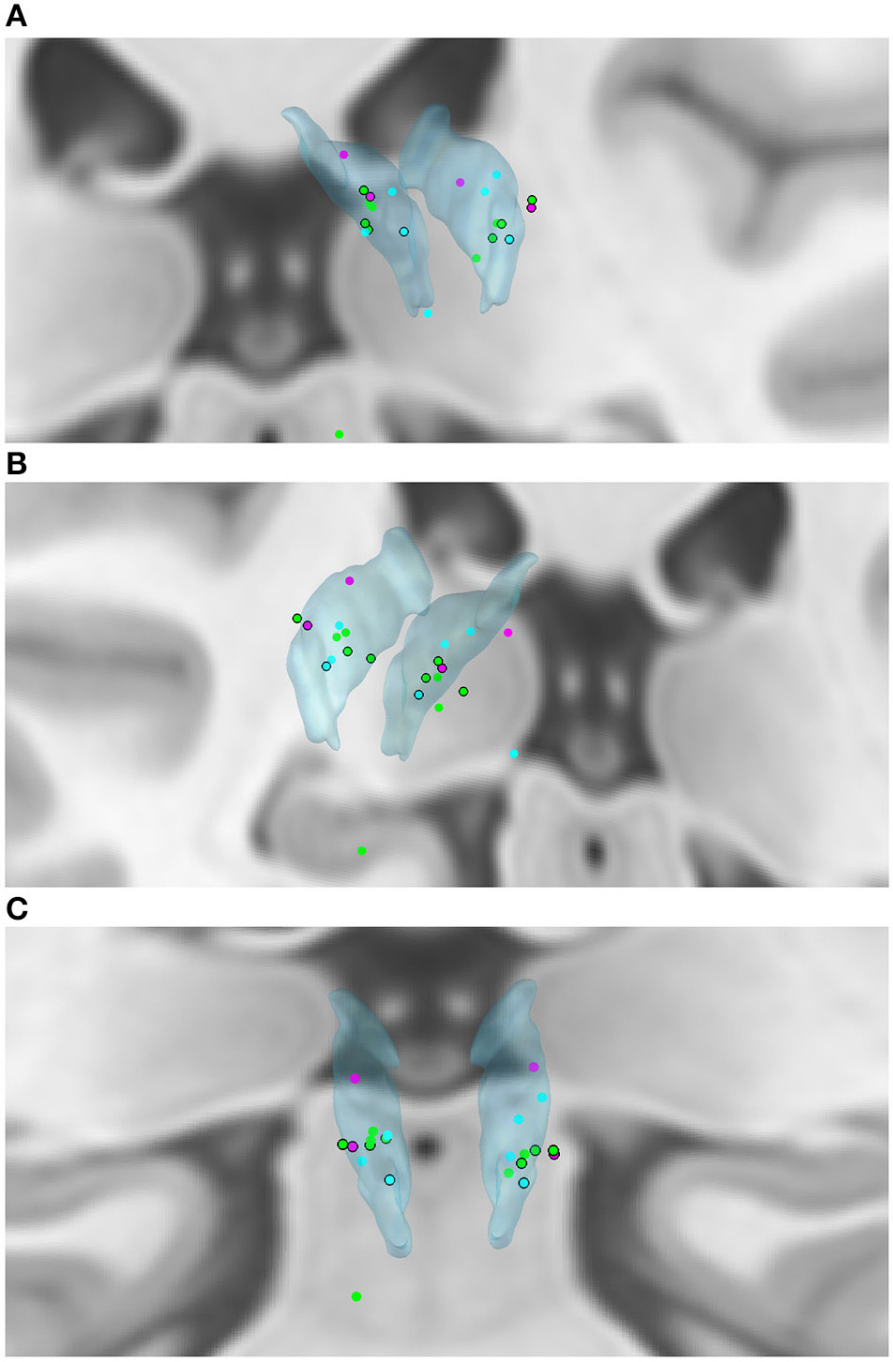
**(A–C)** Active electrode placements in the ANT (translucent blue). Blue dot—seizure reduction < 50%; Green dot—seizure reduction ≥50%; Pink dot—seizure increase; Black ring around dot—intolerable side effects. Seizure outcomes displayed are averages over follow-up.

We created a model of electrode placement using the Lead-DBS toolbox for MatLab (14) (Figures 2A–C) with the DISTAL atlas for 3D visualization (15, 16). Eight of twenty-two active electrodes (36.4%) were outside of the ANT, both electrodes in two patients and one electrode in four patients. Only two of these patients had intolerable side effects (Figures 2A–C, black-ringed dots).

Both patients with seizure increase had one active electrode outside of the ANT (the right electrode in both cases). The 4 remaining patients had seizure decreases of 43.3–86.3%. Two of these four patients had both electrodes off-target.

### Cognitive effects

Data on cognitive effects was complete in 8 of the 13 patients.

#### Acute cognitive effects of DBS

At baseline, i.e., after implantation and before stimulation, 4 of the 8 patients showed impairment in attention and executive functions (1 mild, 3 severe; no floor effects). Under DBS, the one patient with mild impairment significantly deteriorated to a severely impaired level, the other 7 were unchanged according to RCIs. Regarding episodic memory, in 6 of the 8 patients a deficit was registered at baseline (3 mild, 3 severe; no floor effects). Although we did not observe any statistically significant intraindividual memory changes under DBS, there were some categorical changes, i.e., under DBS all patients showed an impairment (4 mild, 4 severe).

#### Long-term effects of DBS on cognition

To address the long-term effects of DBS on cognition we compared the preoperative neuropsychological profile with a long-term follow-up under DBS. The median interval between DBS implantation and follow-up assessment was 54.5 weeks. The preoperative cognitive profile of the 8 patients indicated deficits in attention and executive functions in 6 patients (2 mild, 4 severe), in verbal memory in 7 patients (2 mild, 5 severe), in figural memory in 6 patients (6 severe), in confrontative naming in 8 patients (1 mild, 7 severe), and in mental rotation in 3 patients (3 mild). Data did not indicate relevant floor effects that may have masked subsequent (significant) deteriorations. At the long-term follow-up, we observed significant intraindividual changes in verbal learning and memory in 5 of the 8 patients (3 deteriorated, 2 improved). In detail, 1 patient significantly declined in verbal learning and memory performance, 1 patient in verbal learning and recognition performance, and 1 in absolute delayed free recall. Regarding figural memory, none of the patients declined, 1 patient improved. The same is valid for attention and executive functions (1 improvement), confrontative naming (1 improvement, 2 missing), and mental rotation (1 improvement, 1 missing).

## Discussion

In our cohort, 81.8% of patients treated with ANT-DBS for refractory epilepsy experienced seizure reduction, with an average seizure reduction during entire follow-up of 58.7% (36.5–94.9%). Two patients (18.2%) suffered an average seizure increase of 27.3% during entire follow-up, with a period of significant seizure increase at 12 months. Intolerable side effects arose in 5 patients, mostly psychiatric in nature, and most commonly the emergence of functional neurological disorders.

In our cohort, several patients with multifocal epilepsies benefit from ANT-DBS, in accordance with other centers' experiences (17). Patients with genetic generalized epilepsies may also benefit from DBS. Psychiatric side effects were more common in our cohort than in other published cohorts, and were occasionally severe enough to entail explantation. This may be explained by the fact that more than a third of our implanted electrodes were off-target, compared to approximately 10% in SANTE (1, 2). It is also relevant that, at the time of implantation, usage of electrode model Medtronic 3387 was widespread. In the years of its use, the 3387 electrode had 1.5 mm spacing, with fewer contacts in ANT, compared to the current electrode with 0.5 mm spacing. Additionally, it was not known at that time that it was optimal to target the region of termination of the mammillothalamic tract. Both patients with seizure increase had one active electrode outside of the ANT (the right electrode in both cases). Interestingly, the 4 remaining patients (two of whom had both electrodes off-target) had seizure decreases of 43.3% to 86.3%. This may be due to a variety of reasons: firstly, stimulation in the ANT or in close proximity may be similarly effective. Secondly, the modeling of electrode placement using software cannot be expected to be 100% accurate. Thirdly, interindividual anatomical variation of the exact placement of the ANT may pose a challenge for neurosurgical targeting (18). Furthermore, published data support the hypothesis that proximity to the ANT alone does not correlate with seizure reduction in ANT-DBS, whereas proximity to the mammillothalamic junction does (19). The electrode placement of patients with intolerable side effects seemed to form a cluster in the anterolateral segment of the ANT (Figures 2A–C, black-ringed dots). Interestingly, patients with ≥50% seizure reduction similarly seemed to cluster in a narrow band of the mid- to anterior segment of the ANT (Figures 2A–C, green dots).

Wound-related side effects including paresthesias occurred in our cohort and seem to be among the most common undesirable outcomes of DBS, as described in the SANTE studies (1, 2). It is now known that they usually result from use of the stimulator case as the anode, and that if turning down the current does not relieve the paresthesias, then switching to bipolar stimulation with the stimulator and extension leads taken out of the circuit usually does (1, 2).

Regarding the acute cognitive effects of DBS, only 1 of the assessed patients showed a statistically significant deterioration in executive functions. Although there were no significant changes in verbal memory, 2 patients showed a de novo deficit after a non-significant decline. Long-term neuropsychological effects included significant intraindividual deteriorations as well as improvements in verbal learning and memory. Figural memory, attention and executive functions, confrontative naming and mental rotation were mostly unchanged, and improved in few cases. Though these findings are meaningful, pinpointing their exact cause is challenging: several factors may be at play, such as stimulation programming, stimulation site, and the effect of seizure reduction on cognition, among others.

Due to lack of high-level evidence, there are currently no available standardized treatment guidelines for ANT-DBS with detailed evidence-based stimulation settings. Nevertheless, recently a European expert-panel consensus paper and an international consensus paper (20, 21) issued a series of recommendations and causes for concern, as well as experience-based opinions on the implementation of ANT-DBS. The majority of the panel agreed on broad aspects of stimulation settings (initial monopolar stimulation, most parameters according to the SANTE study). Currently, two main aspects seem decisive, but uncertain, in the effectiveness of ANT-DBS in published works (5, 17, 22–25): patient recruitment (more specifically etiology of epilepsy), and optimal stimulation settings. One of the largest single-center cohorts of patients treated with ANT-DBS (22) followed a systematic approach beginning with voltages under 5V and with minimal medication changes, and reported a responder rate of 73.9%. Lower voltages are coupled with decreased risk of side effects and longer battery life, though patients with higher impedances may need higher amplitudes. When deciding whether to apply monopolar or bipolar stimulation, it is important to consider that monopolar settings result in a wider range of stimulated tissue. When this is coupled with higher voltage, adverse reactions may arise.

Five patients in our cohort had undergone VNS. One patient received concomitant stimulation from the VNS and ANT-DBS. Though initially, it was common practice to require deactivation and/or removal of the VNS system before proceeding with ANT-DBS, recent data shows that here were no complications related to concomitant VNS and ANT-DBS, and removal of VNS does not appear to be necessary (26). Since ANT-DBS and VNS affect seizure control through different mechanisms, concomitant implantation may even be beneficial in certain patients.

Our study is limited by the small sample size and the heterogeneity in patient characteristics. This rendered subgroup analyses uninformative. Though we have strived to offer a more complete picture of life after implantation of DBS by including neuropsychological and side effect outcomes, a more nuanced approach including sleep disruption, subjective impact on quality of life, etc. is needed. Furthermore, all epilepsy studies based on patient-reported seizure frequencies probably suffer from seizure under-reporting (27), and ours is no exception.

We confirm that we have read the Journal's position on issues involved in ethical publication and affirm that this report is consistent with those guidelines.

## Data availability statement

The raw data supporting the conclusions of this article will be made available by the authors, without undue reservation.

## Ethics statement

Ethical review and approval was not required for the study on human participants in accordance with the local legislation and institutional requirements. Written informed consent for participation was not required for this study in accordance with the national legislation and the institutional requirements.

## Author contributions

KO: data curation (lead), data analysis (lead), statistical analysis (lead), MATLAB graphics (lead), writing—original draft preparation (lead), and writing—review and editing (equal). J-AW: neuropsychological data curation (lead), neuropsychological data analysis (lead), writing—original draft preparation (equal), and writing—review and editing (equal). RW: data curation (supporting) and writing—review and editing (equal). CH: supervision (equal), neuropsychological data analysis (supporting), writing—original draft preparation (supporting), and writing—review and editing (supporting). RS: supervision (equal), writing—original draft preparation (supporting), and writing—review and editing (equal). All authors contributed to the article and approved the submitted version.
